# Evaluation of Metabolic Changes in Acute Intermittent Porphyria Patients by Targeted Metabolomics

**DOI:** 10.3390/ijms23063219

**Published:** 2022-03-16

**Authors:** Alex Gomez-Gomez, Paula Aguilera, Klaus Langohr, Gregori Casals, Cristina Pavon, Josep Marcos, Jordi To-Figueras, Oscar J. Pozo

**Affiliations:** 1 Applied Metabolomics Research Group, IMIM, Hospital del Mar, Doctor Aiguader 88, 08003 Barcelona, Spain; agomez@imim.es; 2 Integrative Pharmacology and Systems Neuroscience Group, IMIM, Hospital del Mar, Doctor Aiguader 88, 08003 Barcelona, Spain; klangohr@imim.es; 3 Department of Medicine and Life Sciences (CEXS-UPF), University Pompeu Fabra, Doctor Aiguader 88, 08003 Barcelona, Spain; jose.marcos@anapathresearch.com; 4 Diagnosis and Management of Porphyria Clinic, Department of Dermatologyt, Hospital Clinic, University of Barcelona, Villarroel 170, 08036 Barcelona, Spain; paguile@clinic.ub.es; 5 Department of Statistics and Operations Research, Universitat Politècnica de Barcelona Barcelonatech, 08034 Barcelona, Spain; 6 Biochemistry and Molecular Genetics Unit, Hospital Clinic, IDIBAPS, University of Barcelona, Villarroel 170, 08036 Barcelona, Spain; casals@clinic.cat (G.C.); jto@clinic.cat (J.T.-F.); 7 Cerba Internacional, Pl. Ramon Llull 7-10, 08203 Sabadell, Spain; cristina_pavon@waters.com

**Keywords:** acute intermittent porphyria, metabolomics, LC-MS/MS, tricarboxylic acid cycle, tryptophan, kynurenine

## Abstract

Acute intermittent porphyria (AIP) is an inherited rare hepatic disorder due to mutations within the hydroxymethylbilane gene. AIP patients with active disease overproduce aminolevulinic acid (ALA) and porphobilinogen (PBG) in the liver which are exported inducing severe neurological attacks. Different hepatic metabolic abnormalities have been described to be associated with this condition. The goal of this research was to explore the metabolome of symptomatic AIP patients by state-of-the art liquid chromatography-tandem mass spectrometry (LC-MS/MS). A case versus control study including 18 symptomatic AIP patients and 33 healthy controls was performed. Plasmatic levels of 51 metabolites and 16 ratios belonging to four metabolic pathways were determined. The results showed that the AIP patients presented significant changes in the two main areas of the metabolome under study: (a) the tryptophan/kynurenine pathway with an increase of tryptophan in plasma together with increase of the kynurenine/tryptophan ratio; and (b) changes in the tricarboxylic acid cycle (TCA) including increase of succinic acid and decrease of the fumaric acid/succinic acid ratio. We performed a complementary in vitro study adding ALA to hepatocytes media that showed some of the effects on the TCA cycle were parallel to those observed in vivo. Our study confirms in plasma previous results obtained in urine showing that AIP patients present a moderate increase of the kynurenine/tryptophan ratio possibly associated with inflammation. In addition, it also reports changes in the mitochondrial TCA cycle that, despite requiring further research, could be associated with an energy misbalance due to sustained overproduction of heme-precursors in the liver.

## 1. Introduction

Acute intermittent porphyria (AIP) is an inherited rare hepatic disorder caused by a defect in porphobilinogen deaminase (PBGD, EC 2.5.1.61), the third enzyme of the heme biosynthesis pathway [1]. The expression of AIP is characterized by acute neurovisceral attacks, provoked by the hyperactivity of δ-aminolevulinic synthase (ALAS-1) [2]. The ALAS-1 is the first enzyme of the heme biosynthetic pathway that catalyzes the formation of δ-aminolevulinic acid (ALA) from succinyl-CoA and glycine. The combination of ALAS-1 overexpression with a PBGD deficiency induces to an overproduction of ALA and porphobilinogen (PBG). Since ALA is known to be neurotoxic, its accumulation is associated with the acute crisis but the etiopathogenesis of most neurological manifestations of AIP remains unknown. 

Acute neurovisceral attacks are usually treated by hemin (heme-arginate) intravenous injections [3,4]. Injections restored hepatic heme and decrease ALAS1 overexpression through a feedback mechanism [5]. Recently givosiran (Givlaari, Alnylam Pharmaceuticals), a small silencing RNA (siRNA), has been shown to effectively decrease mALAS1 mRNA levels, preventing the ALA/PBG accumulation and reducing acute attacks incidence [6,7,8].

The molecular mechanisms beneath the recurrence of neurovisceral attacks in some symptomatic AIP patients, however, are not fully understood. The evaluation of the genome in combination with the metabolome alterations produced by AIP might shed light on these mechanisms [9,10,11]. Several metabolic alterations produced by AIP have been previously reported [12,13,14,15,16,17,18]. For instance, steroids are incriminated in the induction of porphyria [19] and our group reported that AIP alterations in the urinary steroidome are correlated with ALA concentration [15,16]. Misbalances in amino acids such as glycine [20], homocysteine [21], tryptophan (Trp), and long neutral amino acids (LNAAs) have also been reported [12,22]. Some of these metabolic alterations, e.g., the increase of homocysteine levels, have been reported to be aggravated by givosiran administration [3,23]. In addition, increased urinary levels in the precursor metabolic pathways of the heme biosynthesis and tricarboxylic acid cycle (TCA) have also been reported in asymptomatic AIP patients [14] as well as animal models have been used to determine the TCA energetic failure in the pathophysiology of AIP [24,25,26]. Despite the accumulated knowledge in metabolic alterations produced by AIP, these studies usually dealt with a limited part of the metabolome determined in heterogeneous cohorts. Thus, the evaluation of a broad part of the metabolome in samples collected in the active phase of the disease from a homogeneous cohort would be more valuable.

Facing issues such as the chemical complexity and heterogeneity of the samples, the wide dynamic range and the expensive cost of the analysis [27], metabolomics is the option of choice for determining metabolic changes promoted by a pathological status [28]. Although untargeted approaches can provide global information about potential alterations produced by the disease, their main limitations are related with both the determination of low concentrated metabolites and the proper quantification of metabolites. Targeted metabolomics is an alternative that minimize these two drawbacks while presenting several other advantages such as (i) the capability to extract information by the analyses of a large number of samples from different batches or (ii) the provision of accurate information and the possibility of in deep study of the selected metabolic pathways [29]. 

Hence, the main aim of this study was to evaluate metabolomic changes produced by AIP patients in the active phase of the disease. For that purpose, plasma samples from cases and controls were analyzed in order to investigate four different metabolic pathways. Metabolites from tryptophan metabolism, TCA, amino acids and steroidogenesis were determined by four specific liquid chromatography-tandem mass spectrometry (LC-MS/MS) methods. 

## 2. Results

### 2.1. Targeted Metabolomics Is Able to Differentiate AIP Cases and Controls

The four different targeted LC-MS/MS methods provide information about 51 metabolites belonging to the following metabolic pathways: (a) Trp metabolism, (b) TCA cycle, (c) amino acids, and (d) steroids. Additionally, 16 different ratios between the quantified metabolites were quantified since they provide information about the enzymatic activity involved in the different pathways.

**Figure 1:**
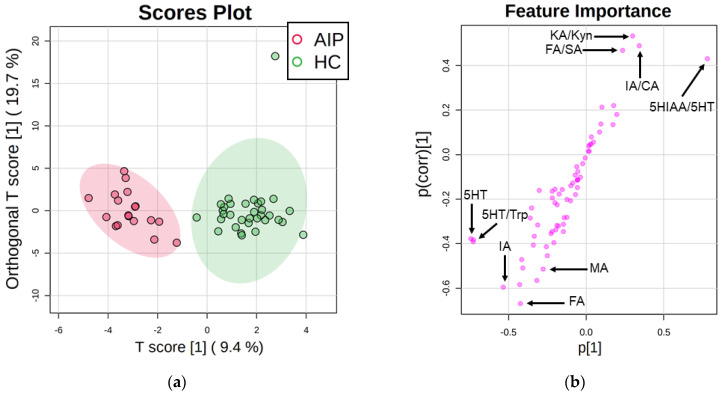
OrthoPLS-DA analysis. Comparison between HC and AIP cohorts. (**a**) Scores plot of orthoPLS-DA and (**b**) signature feature. Plots of the orthoPLS-DA reveal clear separation between groups and the altered metabolic pathways (Trp and TCA cycle). Abbreviations: Kynurenic acid/kynurenine (KA/Kyn); fumaric acid/succinic acid (FA/SA); isocitric acid/citric acid (IA/CA); 5-hydroxyindoleacetic acid/serotonin (5HIAA/5HT); serotonin (5HT); serotonin/tryptophan (5HT/Trp); isocitric acid (IA); malic acid (MA); fumaric acid (FA).

The orthogonal partial least square-discriminant analysis (orthoPLS-DA) demonstrated the suitability of the selected metabolic pathways to separate healthy control (HC) and AIP groups (Figure 1a). Signature features showed that the most altered biomarkers in AIP patients belonged to Trp metabolism (e.g., kynurenic acid/kynurenine (KA/Kyn) ratio) and TCA cycle (e.g., isocitric acid/citric acid (IA/CA) ratio) (Figure 1b). In contrast, steroid biomarkers and amino acids were not among the most altered biomarkers in AIP. For this reason, the present research was focused on the evaluation of AIP disturbances in the tryptophan metabolism and TCA cycle.

### 2.2. Tryptophan Metabolism

Plasmatic concentrations of Trp and six of its metabolites together with seven ratios belonging to that pathway were calculated (Table 1). Most biomarkers were adjusted to the model after logarithm transformation (Table 1). Additionally, 5HIAA and Kyn metabolites and Kyn/Trp and KA/Kyn and 3OHKyn/Kyn ratios were not adjusted to the model even after logarithm transformation and their results were obtained by T-Welsh.

**Figure 2:**
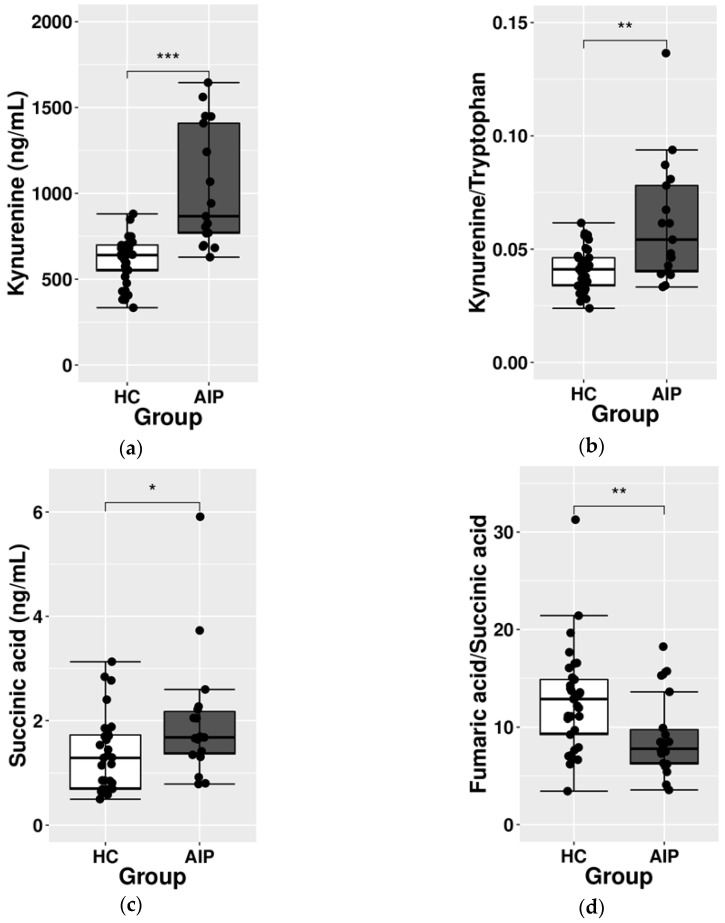
Boxplots showing differences between HC and AIP patients. Representation of (**a**) kynurenine, (**b**) kynurenine/tryptophan, (**c**) succinic acid, and (**d**) fumaric acid/succinic acid. Asterisks code: * *p*-value between 0.01–0.05; ** *p*-value between 0.001–0.01; *** *p*-value < 0.001. Abbreviation: HC: healthy controls, AIP: acute intermittent porphyria.

Among the selected Trp markers, several of them were altered in AIP (Table 1). Thus, AIP patients showed a significant increase in plasmatic Trp levels (*p* = 0.023). Both metabolic pathways belonging to tryptophan metabolism (serotonin and kynurenine pathways) were found to be altered in AIP. On the one hand, 5HT, 5HIAA, 5HT/Trp, and 5HIAA/Trp (all belonging to the serotonin pathway) were significantly increased in AIP (*p* < 0.01 in all cases) whereas 5HIAA/5HT decreased in AIP (*p* = 0.005) suggesting a 5HT accumulation. On the other hand, we found significant alterations in several markers belonging to the kynurenine pathway. Thus, Kyn showed a significant increase in AIP patients (*p* < 0.001, Figure 2a). Similarly, all the ratios including Kyn were altered in AIP: Kyn/Trp was increased in AIP (*p* = 0.003, Figure 2b) whereas KA/Kyn (*p* < 0.001) and 3OHKyn/Kyn (*p* = 0.011) were decreased in AIP.

### 2.3. TCA Cycle

Five different TCA intermediates and five ratios between them were determined in plasma and included in the analysis (Table 1). Most metabolites were adjusted to the model after logarithm transformation. CA metabolite and MA/FA and CA/MA ratios required the application of T-Welsh.

Results revealed an upregulation of the TCA cycle in AIP patients (Table 1). A significant increment from 2- to 4-fold was found in IA, FA, MA, and SA (*p* < 0.001 for IA, FA, and MA and *p* = 0.019 for SA) (Figure 2c,d). In addition, the enzymatic interconversion of CA into IA (IA/CA ratio) was significantly decreased in AIP (*p* < 0.001) as well as the FA/SA ratio (*p* = 0.007) while SA/IA and MA/FA ratios were increased in AIP patients (*p* = 0.013 and *p* = 0.025, respectively).

### 2.4. Increase in TCA Metabolites after Addition of ALA to Hepatocytes Culture

**Figure 3:**
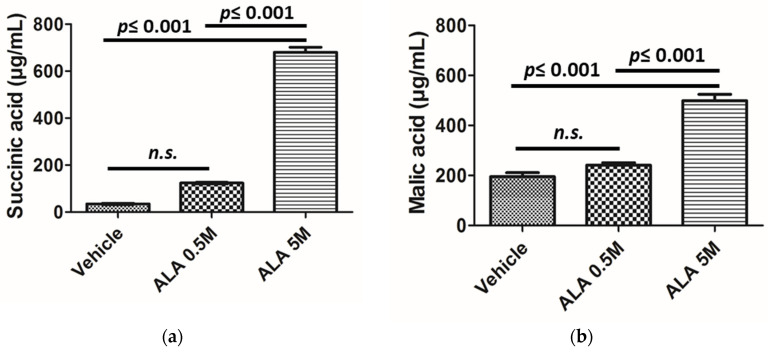
Effect of ALA addition into human hepatocytes metabolism. Production of (**a**) succinic acid by hepatocytes after addition of vehicle, 0.5 of ALA and 5 of ALA, and (**b**) malic acid by hepatocytes after addition of vehicle, 0.5 of ALA and 5 of ALA. Abbreviations: ALA: δ-aminolevulinic acid, n.s.: no significant.

Hepatocytes cultures with ALA at different concentrations showed an increase in the levels of TCA metabolites. This increase was found to be more remarkable in SA. The addition of ALA 0.5-M raised the SA concentrations 3.5 times whereas a 29-fold increment was observed after addition of ALA 5M (Figure 3). The remaining TCA metabolites also showed higher concentrations after ALA addition, but their increase was less pronounced than for SA. Remarkably, the rise in concentrations decreased following TCA cycle being SA and FA the ones with higher increases and CA an IA the ones with less pronounced rises.

## 3. Discussion

Despite the great potential of metabolomics, its application in rare diseases is limited. Due to the inherent nature of rare diseases, obtaining an appropriate number of samples to perform metabolomics studies is hard and sometimes requires long periods of time. As an example, in the present study 18 AIP samples were collected over a two-year period. In order to minimize the potential bias added by the storage period, both HC and AIP samples were collected over the same period. In addition, we excluded from the study those analytes that are known to be unstable in plasma samples over time (e.g., lactic acid, pyruvic acid, etc.) following the “The Quality of Diagnostic Samples” guideline [47]. Finally, 67 plasmatic biomarkers (51 analytes and 16 ratios) were included in the study. Results obtained from selected metabolites in healthy controls were in accordance with the previously reported range in literature [30,31,32,33,34,35,36,37,38,39,40,41,42,43,44,45,46] (Table 1).

**Figure 4:**
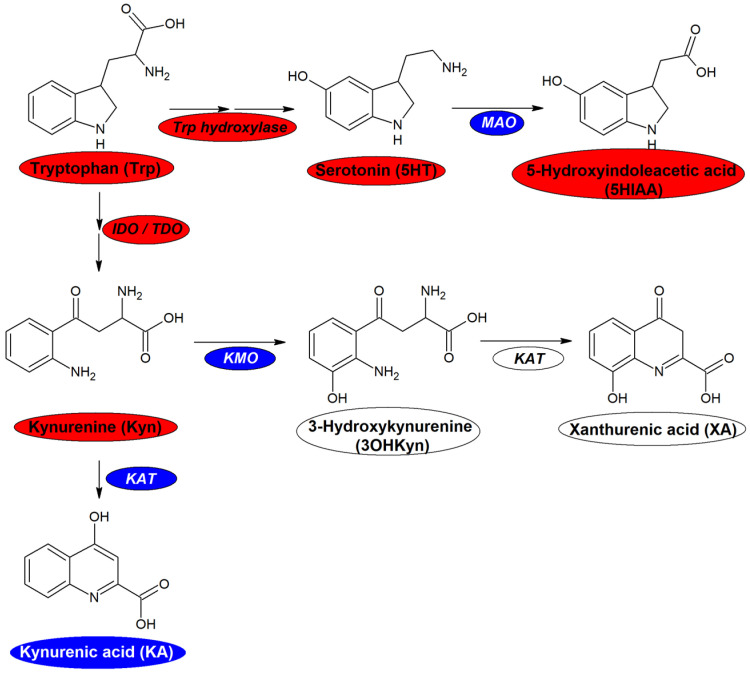
Tryptophan metabolism. Abbreviations: MAO: monoamine oxidase, IDO: indoleamine 2,3-dioxygenase, TDO: tryptophan 2,3-dioxygenase, KMO: kynurenine 3-monooxygenase, KAT: kynurenine aminotransferase. Color code: blue: decreased in AIP patients, white: unaltered in AIP patients, red: increased in AIP patients.

Our results suggest that AIP patients present alterations in several metabolic pathways. First, we found changes in Trp metabolism. AIP patients presented a moderate increase of plasmatic Trp and 5HT. These findings are in agreement with a previous report [36] that hypothesized that during acute attacks of porphyria heme-deficiency induce a decrease of in Trp 2,3-dioxygenase (TDO), the main enzyme that converts Trp into Kyn in the liver (Figure 4) [42]. Metabolism of tryptophan to kynurenine is catalyzed by two different enzymes: heme-dependent TDO operating in the liver and IDO in all tissues. TDO may be sensible to hepatic heme fluctuations, thus decreasing Trp metabolism to Kyn although IDO is activated by inflammation. In addition, we also found an increase in the Kyn/Trp ratio in plasma, thus confirming our previous findings in urine [12]. The observed changes may be due to both a moderate TDO decrease in the liver and upregulation of extra-hepatic IDO due to inflammation [48]. Altogether, AIP patients seem to overproduce several key metabolites belonging to both serotonin and kynurenine pathways.

**Figure 5:**
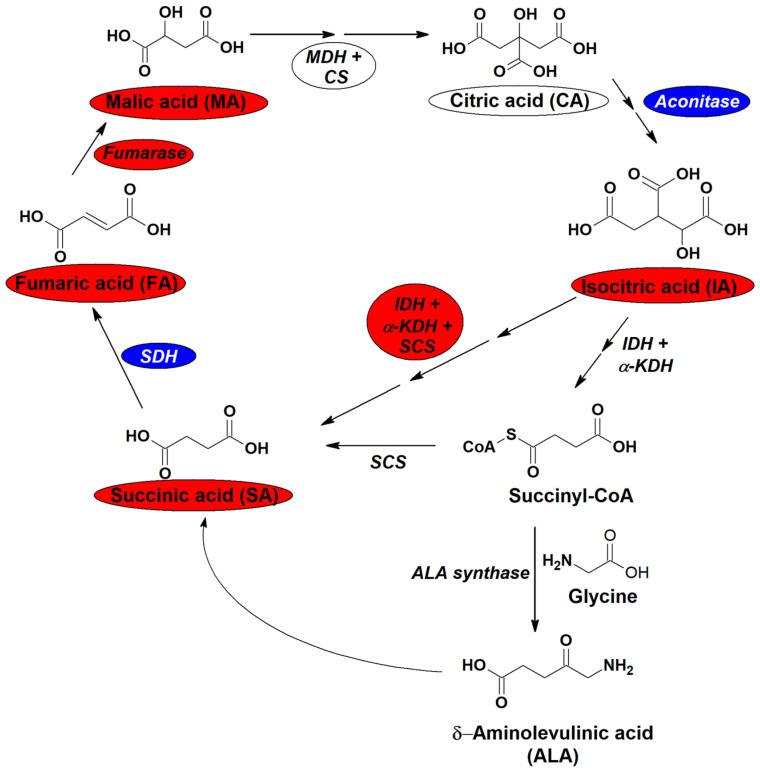
TCA cycle. Abbreviations: LDH: lactate dehydrogenase, IDH: isocitrate dehydrogenase, α-KDH: α-ketoglutarate dehydrogenase, SCS: succinyl Co-A synthetase, SDH: succinate dehydrogenase, MDH: malate dehydrogenase, CS: citrate synthase. Color code: blue: decreased in AIP patients, white: unaltered in AIP patients, red: increased in AIP patients.

Plasmatic levels of some TCA metabolites were also modified in AIP patients. Disorders associated to hepatic dysregulation are prone to suffer from TCA disruptions [49]. Our results showed a clear increase (2–4-fold) in AIP patients of the plasmatic levels of most of the TCA intermediates (Figure 5). This overproduction of TCA metabolites could suggest a mitochondrial dysfunction producing an imbalance in energy production associated with active hepatic AIP and overproduction of heme-precursors. Our results, however, are different from those obtained in AIP mouse models in which cataplerosis of TCA was the main finding [26].

Our observation that ALA added to hepatocyte cell culture increase some TCA intermediates in a similar fashion to our in vivo observation is intriguing. It would be optimally explained if ALA was back-converted to SA. However, to the best of our knowledge this back-conversion has not been reported in humans as it has been described in other species [50]. Obtaining SA from ALA would by-pass the succinyl CoA synthetase (SCS), an enzyme directly involved in the obtaining of either ATP or GTP. Therefore, circumventing SCS might impair the energetic production. Thus, further research is needed (using C-labeled intermediates) to prove this hypothesis.

In summary, among the AIP patients and using our metabolome approach we observed an imbalance in serotonin, kynurenine pathways and mitochondrial TCA. The most plausible explanation is that AIP condition itself inducing energy consuming sustained overproduction of ALA/PBG could originate those changes, especially the mitochondrial TCA dysregulation. However, since symptomatic AIP is associated with a low-grade systemic inflammation [51,52] we cannot discard a pro-inflammatory effect inducing, i.e., changes in the tryptophan metabolism.

## 4. Materials and Methods

### 4.1. Patients

Nineteen symptomatic AIP patients (17 women, 2 man) with variable clinical condition were included in the study. All had biochemically active disease (urinary PBG and ALA > 10 nmol/mmol of creatinine) and were followed at the Porphyria Unit of the Hospital Clinic of Barcelona. The AIP diagnosis was based on biochemical analyses of urine, feces, blood, and genetic sequencing of the HMBS gene according to European Porphyria Network (EPNET) (https://porphyria.eu, accessed on 10 June 2021) recommendations. HMBS gene mutations and clinical characteristics of most of these patients have been reported earlier [10,53]. Seven of these patients presented recurrent attacks and were receiving prophylactic heme-arginate infusions every two weeks.

Blood and urine samples were collected from all patients when they attended the porphyria unit for follow-up or to receive prophylactic hemin. Blood sampling was routinely performed between 08.00 and 10.00 after overnight fasting, according to hospital protocols. All patients receiving heme-arginate had samples collected prior to the intravenous infusion. Blood was immediately centrifuged after extraction and plasma stored at −80 °C until analyses.

All subjects gave their informed consent for inclusion before they participated in the study. The study was conducted in accordance with the Declaration of Helsinki, and the protocol was approved by the Ethics Committee of Ethics (CEIC; “Comité Ético de Investigación Clínica”) of the IMIM Hospital del Mar (2014/5802/I).

### 4.2. Standards and Reagents

The standards and reagents used in the present research were bought differently in Sigma Aldrich (Saint Louis, MO, USA), Alsachim (Illkirch-Graffenstaden, France), Toronto Research Chemicals (North York, ON, Canada), Cambridge Isotope Laboratories (Tewksbury, MA, USA), Merck (Darmstadt, Germany), NMI (Sydney, Australia), and Millipore Ibérica (Barcelona, Spain). A summary is represented in Supplementary Table S1.

### 4.3. Quantification of Plasmatic Biomarkers

The quantification of the 51 plasmatic biomarkers and 16 ratios between them was achieved by using 4 different LC-MS/MS methods. Differences in the chemical structure of the analytes made necessary the application of different analytical approaches for the proper quantification of the analytes.

#### 4.3.1. Tryptophan Metabolism

Biomarkers belonging to tryptophan metabolism were quantified following a previously reported method [54]. Briefly, 50 µL of the internal standard solution (ISTD) were added to 100 µL of plasma. The protein precipitation achieved by the addition of 300 µL of acetonitrile was followed by a vigorous vortex shaking and a centrifugation step. The liquid layer was then evaporated until dryness (N2 stream, 29 °C, <15 psi) and then reconstituted with 100 µL of water. Finally, 10 µL were injected into the LC-MS/MS instrument.

#### 4.3.2. TCA Intermediates

The analysis of TCA intermediates was performed by using a previously reported method [55]. Initially, the method consisted in a previous dilution of plasma (10-fold) and 10 µL of the dilution were employed for the analysis. Subsequently, 30 µL of the ISTD were added and the sample derivatization was performed by the addition of 100 µL of the mixture of o-benzyl hydroxylamine (1 M) and N-(3-Dimethylaminopropyl)-N′-ethylcarbodiimide hydrochloride (1 M) during 1 h at room temperature. Then, a liquid–liquid extraction with water and ethyl acetate was performed and the organic extract was evaporated until dryness (N2 stream, 40 °C, <15 psi). The extracts were reconstituted in 150 µL of water:methanol (1:1) and 10 µL were injected into the LC-MS/MS system.

#### 4.3.3. Amino Acids

The analysis of amino acids was carried out by employing a combination of AccQ-Tag chemical derivatization with LC-MS/MS employing the procedure described elsewhere [56]. Briefly, 50 μL of either a standard amino acid mix solution, or a biological extract were vortex with 100 μL of acetonitrile, to precipitate proteins. Ten μL of the resultant supernatant was mixed with 70 μL of AccQ-Tag Ultra borate buffer, and 20 μL of AccQ-Tag reagent. The reaction was allowed to proceed for 10 min at 55 °C. Two µL of the final extract was injected in the LC-MS/MS system.

#### 4.3.4. Steroids

Steroids were determined by the adaptation of a previously reported method [57,58]. The analysis used 100 µL of plasma and 50 µL of ISTD were added. Then, a liquid–liquid extraction with NaCl(s), K_2_CO_3_ and ethyl acetate was performed, and the organic extract was evaporated until dryness (N2 stream, 40 °C, <15 psi). The extracts were then reconstituted in 100 µL of water:methanol (1:1) and 10 µL were injected into the LC-MS/MS system.

### 4.4. In Vitro Experiments

A HepG2 cells were seeded (1 × 10^6^ cells) in T75 flasks and grown to confluence in DMEM, supplemented with 10% fetal bovine serum, 50 U/mL of penicillin, and 50 μg/mL of streptomycin. Cells were placed in a water-jacketed CO_2_ incubator (37 °C, 5% CO_2_) (Nuaire from Plymouth, MN, USA). After reaching confluence, cells were switched to serum-free medium for 16 h. Then, cells were subsequently incubated in 10 mL of HBSS containing ALA (0.5 and 5 M in PBS) or vehicle (50 µL of PBS). Supernatants (0.5 mL) were obtained from the same ALA-treated or vehicle-treated flask after an incubation period of 24 h. After collection, supernatants were centrifuged at 670× *g* (4 °C) and frozen at −20 °C until further analysis. Hepatic cells were observed by optical microscopy before and after completion of each incubation period. No differences in cell integrity and morphology were observed between cells exposed to ALA or vehicle throughout the incubation periods. No detachment of cells was observed after incubation in all conditions.

### 4.5. Instrumentation

The instrumentation was carried out by using an Acquity UPLC system coupled to a triple quadrupole (Xevo TQs) mass spectrometer (Waters Associates, Milford, MA, USA) provided with an orthogonal Z-spray-electrospray interface (ESI).

The LC separation for tryptophan metabolites, TCA and steroids was performed using an Acquity BEH C18 column (100 × 2.1 mm i.d., 1.7 µm) (Waters Associates) with a flow rate of 300 µL/min at 55 °C. In the case of amino acids, a CORTECS UPLC C18 column (150 × 2.1 mm i.d., 1.6 µm) (Waters Associates) with a flow rate of 500 µL/min at 55 °C was used. For the MS detection, positive ionization mode was selected for all methods.

Mobile phases selected for the determination of tryptophan metabolites, TCA, and steroids was water–ammonium formate (1 mM)–formic acid (0.01%) as mobile phase A and methanol–ammonium formate (1 mM)–formic acid (0.01%) as mobile phase B. However, a specific chromatographic gradient was employed in each analytical method. The gradient program for the determination of tryptophan metabolites increased linearly the percentage of mobile phase B as follows: 0, 1%; 0.5, 1%; 7, 40%; 8.5, 90%; 9, 90%; 9.5, 1%; 12 min, 1%. For the TCA analytes determination, the gradient program changes linearly the percentage of mobile phase B as follows: 0, 30%; 1, 30%; 6, 55%; 6.8, 80%; 8.3, 99%; 9, 99%; 9.01, 30%; 10 min, 30%. The determination of steroids was performed by a gradient program with a percentage of mobile phase B linearly changing as follows: 0, 15%; 0.5, 15%; 3, 40%; 16, 70%; 17, 90%; 18, 90%; 18.5, 15%; 20 min, 15%. In the case of amino acids, water–formic acid (0.1%) and acetonitrile–formic acid (0.1%) were selected as mobiles phases A and B, respectively. The gradient program linearly changed the percentage of mobiles phase B as follows: 0, 1%; 1, 1%; 2, 13%; 5.5, 15%; 6.5, 95%; 7.5, 95%; 7.6, 1%; 9 min, 1%.

### 4.6. Statistical Analysis

All data were analyzed using SPSS (IBM Corp. Released 2013. IBM SPSS Statistics for Windows, Version 22.0. Armonk, NY, USA: IBM Corp.), R software (version 4.0.2, https://www.R-project.org), R studio (version 1.2.1335), and Metaboanalyst (version 3.0, https://www.metaboanalyst.ca) software.

Metaboanalyst was used for the multivariate analysis via orthogonal partial least squares discriminant analysis (orthoPLS-DA) followed by a sparse partial least squares discriminant analysis (sPLS-DA). For the multivariate analysis samples with >20% of missing values were discarded and the remaining missing values were replaced by a small value (half of the minimum positive value in the original data). In addition, results were normalized by log transformation.

Given the right-skewed distribution of most variables, the data were log-transformed prior to the inferential analyses. The model assumptions (homoscedasticity and normally distributed residuals) were checked with both the Levene test (for homoscedasticity) and the graphically by means of the QQ plot for normality.

## 5. Conclusions

In the present study, we use targeted metabolomics approaches to report the main metabolic abnormalities associated to AIP. The main features of this imbalance are (i) a dysregulation of Trp metabolism in both serotonin and kynurenine pathways (as previously described in urine) and (ii) a mitochondrial dysfunction. Since AIP induces to the sustained energy consumption for the ALA/PBG overproduction, it is likely that mitochondrial TCA changes may be explained by the AIP condition. However, the association between low-grade systemic inflammation and symptomatic AIP, a pro-inflammatory effect inducing, i.e., changes in the Trp metabolism cannot be discard. In addition, as found in humans, we also report changes in TCA after in vitro addition of ALA to culture hepatocyte cells, suggesting the possibility of ALA back-conversion into SA. However, further research should be performed to confirm this hypothesis.

## Figures and Tables

**Table 1:** Reference values (obtained from [30,31,32,33,34,35,36,37,38,39,40,41,42,43,44,45,46]), quantification of each metabolite and metabolic ratios between different analytes and statistical results (*p*-value and mean difference (95% CI)). Abbreviations: Trp: tryptophan; 5HT: serotonin; 5HIAA: 5-hydroxyindoleacetic acid; Kyn: kynurenine; KA: kynurenic acid; 3OHKyn: 3-hydroxy-kynurenine; XA: xanthurenic acid; CA: citric acid; IA: isocitric acid; SA: succinic acid; FA: fumaric acid; MA: malic acid.

**Tryptophan Metabolism**					
**Marker**	**Reference Values (ng/mL)**	**HC (*n* = 33) (ng/mL)**	**AIP (*n* = 18) (ng/mL)**	***p*-Value**	**Mean Difference (95% CI)**
Trp	7557–19,177	15,866 (8911–22,358)	17,891 (12,054–23,566)	0.023	−2184 (−4059–−308)
5HT	0.60–220	25 (0.40–153)	143 (1.5–657)	<0.001 ^a^	−0.78 (−1.14–−0.4) ^a^
5HIAA	1.8–19	11 (5.7–24)	18 (10–48)	<0.001 ^b^	−0.22 (−0.318–−0.12) ^b^
Kyn	146–625	640 (334–880)	867 (629–1645)	<0.001 ^b^	−0.21 (−0.295–−0.13) ^b^
KA	2.7–25	22 (16–47)	28 (20–61)	0.203	−3.4 (−8.8–1.9)
3OHKyn	3.1–18	17 (9.5–38)	19 (7.2–35)	0.786	−0.75 (−6.3–4.8)
XA	0.84–39	66 (11–88)	62 (46–93)	0.497	−3.8 (−15.0–7.4)
5HT/Trp	-	1.5 × 10^−3^ (1.0 × 10^−4^–9.6 × 10^−3^)	8.0 × 10^−3^ (1.0 × 10^−4^–3.7 × 10^−2^)	<0.001 ^a^	−0.72 (−1.08–−0.4) ^a^
5HIAA/Trp	-	7.0 × 10^−4^ (3.0 × 10^−4^–1.6 × 10^−3^)	1.0 × 10^−3^ (6.0 × 10^−4^–3.2 × 10^−3^)	0.004 ^a^	−0.16 (−0.260–−0.05) ^a^
5HIAA/5HT	-	0.51 (0.080–34.0)	0.13 (0.023–1.5 × 10^1^)	0.005 ^a^	0.56 (0.18–0.94) ^a^
Kyn/Trp	-	4.1 × 10^−2^ (2.4 × 10^−2^–6.2 × 10^−2^)	5.4 × 10^−2^ (3.31 × 10^−2^–1.4 × 10^−1^)	0.003 ^b^	−0.15 (−0.249–−0.06) ^b^
KA/Kyn	-	3.7 × 10^−2^ (2.4 × 10^−2^–1.2 × 10^−1^)	2.9 × 10^−2^ (1.8 × 10^−2^–4.5 × 10^−2^)	0.001 ^b^	0.16 (0.073–0.243) ^b^
3OHKyn/Kyn	-	2.8 × 10^−2^ (1.7 × 10^−2^–5.5 × 10^−2^)	2.0 × 10^−2^ (9.4 × 10^−3^–4.7 × 10^−2^)	0.011 ^b^	0.17 (0.044–0.297) ^b^
XA/3OHKyn	-	4.5 (1.1–7.8)	3.8 (1.3–7.3)	0.957 ^a^	−0.0041 (−0.156–0.15) ^a^
**Tricarboxylic Acid Cycle**					
**Marker**	**Reference Values (ng/mL)**	**HC (*n* = 33) (ng/mL)**	**AIP (*n* = 18) (ng/mL)**	***p*-Value**	**Mean Difference (95% CI)**
CA	5.7–77	25 (9.4–38)	30 (5.8–67)	0.211 ^b^	−0.087 (−0.228–0.05) ^b^
IA	>0.20–1.9	1.1 (0.15–2.9)	2.2 (1.1–5.7)	<0.001 ^a^	−0.35 (−0.482–−0.22) ^a^
SA	0.70–3.8	1.3 (0.50–3.1)	1.7 (0.79–5.9)	0.019	−0.65 (−1.188–−0.11)
FA	>0.12–0.46	0.12 (0.022–0.19)	0.22 (0.10–0.44)	<0.001 ^a^	−0.33 (−0.447–−0.21) ^a^
MA	0.31–2.8	1.6 (0.5–3.0)	2.8 (0.8–6.3)	<0.001 ^a^	−0.22 (−0.334–−0.11) ^a^
IA/CA	-	22 (10–62)	14 (5.4–30)	<0.001 ^a^	0.26 (0.150–0.37) ^a^
SA/IA	-	0.7 (0.3–4.7)	1.2 (0.7–3.1)	0.013 ^b^	−0.18 (−0.321–−0.04) ^b^
FA/SA	-	13 (3.4–31)	7.8 (3.6–18)	0.007 ^a^	0.16 (0.044–0.266) ^a^
MA/FA	-	7.2 × 10^−2^ (1.4 × 10^−2^–1.1 × 10^−1^)	8.4 × 10^−2^ (4.4 × 10^−2^–1.3 × 10^−1^)	0.025 ^b^	−0.11 (−0.200–−0.01) ^b^
CA/MA	-	16 (6.0–27)	14 (5.7–26)	0.600 ^b^	0.027 (−0.078–0.132) ^b^

^a^ Results obtained after logarithm transformation. ^b^ Results obtained after logarithm transformation and by T-Welsh. - Reference values not available in the literature.

## Data Availability

The data presented in this study are available in Supplementary Materials.

## Supplementary Materials

The following supporting information can be downloaded at https://www.mdpi.com/article/10.3390/ijms23063219/s1.

## Abbreviations

AIP	Acute Intermittent Porphyria
PBGD	Porphobilinogen Deaminase
ALAS-1	δ-Aminolevulinic Synthase
ALA	δ-Aminolevulinic Acid
PBG	Porphobilinogen
Trp	Tryptophan
LNAAs	Long Neutral Amino Acids
TCA	Tricarboxylic Acid Cycle
LC-MS/MS	Liquid Chromatography-Tandem Mass Spectrometry
HC	Healthy Controls
5HT	Serotonin
KA	Kynurenic Acid
Kyn	Kynurenine
IA	Isocitric Acid
CA	Citric Acid
FA	Fumaric Acid
SA	Succinic Acid
5HIAA	5-Hydroxy Indole Acetic Acid
5HT	Serotonin
MA	Malic Acid
FA	Fumaric Acid
3OHKyn	3-Hydroxy Kynurenine
XA	Xanthurenic Acid
TDO	Tryptophan 2,3-Dioxygenase
SCS	Succinyl CoA Synthetase
ISTD	Internal Standard Solution
