# Supplementary material for: Evaluation of Metabolic Changes in Acute Intermittent Porphyria Patients by Targeted Metabolomics
Source: Int J Mol Sci. 2022 Mar 16;23(6):3219. doi: 10.3390/ijms23063219 (PMC8950560; doi:10.3390/ijms23063219)
Supplement: Supplementary file 1 [file ijms-23-03219-s001.zip › ijms-1613277-supplementary.pdf]

International Journal of *Molecular Sciences*

MDPI

Supplementary material

Evaluation of Metabolic Changes in Acute Intermittent Porphyria Patients by Targeted Metabolomics

Alex Gomez-Gomez [1,2,3], Paula Aguilera [4], Klaus Langohr [2,5], Gregori Casals [6], Cristina Pavon [7], Josep Marcos [3], Jordi To-Figueras [6] and Oscar J. Pozo [1,2,*]

1 Applied Metabolomics Research Group, IMIM, Hospital del Mar, Doctor Aiguader 88, 08003 Barcelona, Spain; agomez@imim.es
2 Integrative Pharmacology and Systems Neuroscience Group, IMIM, Hospital del Mar, Doctor Aiguader 88, 08003 Barcelona, Spain; klangohr@imim.es
3 Department of Medicine and Life Sciences (CEXS-UPF), University Pompeu Fabra, Doctor Aiguader 88, 08003 Barcelona, Spain; jose.marcos@anapathresearch.com (J.M.)
4 Diagnosis and management of Porphyria clinic, Department of Dermatologyt, Hospital Clinic, University of Barcelona, Villarroel 170, Barcelona 08036, Spain; paguile@clinic.ub.es
5 Department of Statistics and Operations Research, Universitat Politècnica de Barcelona Barcelonatech, 08034 Barcelona, Spain
6 Biochemistry and Molecular Genetics Unit, Hospital Clinic, IDIBAPS, University of Barcelona, Villarroel 170, Barcelona 08036, Spain; casals@clinic.cat (G.C.); jto@clinic.cat (J.T.-F.)
7 Cerba Internacional, Pl. Ramon Llull 7-10, 08203 Sabadell, Spain; cristina_pavon@waters.com
* Correspondence: opozo@imim.es; Tel.: +34 93 316 04 80

Table S1. Chemicals and reagents used for the analysis and the comercial source. SA: Sigma Aldrich (Saint Louis, MO, USA); Alsachim (Illkirch-Graffenstaden, France); TRC: Toronto Research Chemicals (Toronto, Canada); CIL: Cambridge Isotope Laboratories (Tewksbury, MA, USA); Merck (Darmstadt, Germany); Steraloids (Newport, RI, USA); NMI: National Measurement Institute (Sydney, Australia); MI: Millipore Ibérica (Barcelona, Spain).

Pathway	Standards and reagents	Commercial Source
Tryptophan metabolism	Tryptophan (Trp)	SA
	Serotonin (5HT)	SA
	5-Hydroxyindoleacetic acid (5HIAA)	SA
	Kynurenine (Kyn)	SA
	Kynurenic acid (KA)	SA
	3-Hydroxy kynurenine (3OHKyn)	SA
	Xanthurenic acid (XA)	SA
	Tryptophan-d5	Alsachim
	Kynurenine-13C6	Alsachim
	3-Hydroxy kynurenine-13C6	Alsachim
	5-Hydroxyindoleacetic acid-d4	Alsachim
	Kynurenine acid-d5	TRC
	Serotonin-d5	TRC

TCA cycle	Lactic acid (LA)	SA
	Pyruvic acid (PyA)	SA
	Citric acid (CA)	SA
	Isocitric acid (IA)	SA
	Succinic acid (SA)	SA
	Fumaric acid (FA)	SA
	Malic acid (MA)	SA
	Lactic acid-13C3	SA
	Pyruvic acid-13C3	TRC
	Citric acid-d4	SA
	Succinic acid-d4	SA
	Fumaric acid-13C4	SA
	Malic acid-d3	CIL
	N-(3-Dimethylaminopropyl)-N'-ethylcarbodiimide hydrochloride	SA
	o-benzyl hydroxylamine	SA
	Hydrochloric acid	Merck
	Pyridine	Merck
Steroids	Cortisol (F)	Steraloids
	20α-dihydrocortisol (20aDHF)	Steraloids
	20β-dihydrocortisol (20bDHF)	Steraloids
	6β-hydroxycortisol (6OHF)	Steraloids
	Cortisone (E)	Merck
	20α-dihydrocortisone (20aDHE)	Steraloids
	20β-dihydrocortisone (20bDHE)	Steraloids
	6β-hydroxycortisone (6OHE)	Steraloids
	Corticosterone (B)	Steraloids
	11-dehydrocorticosterone (A)	Steraloids
	Testosterone (T)	Steraloids
	Androstenedione (AED)	Steraloids
	Progesterone (Prog)	Steraloids
	6β-hydroxycortisol-d4	TRC
	Cortisol-d4	SA
	Testosterone-d3	NMI
	Potassium carbonate	Merck
	Sodium chloride	Merck
Common material	Water milliQ	MI
	Ammonium formate	SA
	Methanol	Merck
	Formic acid	Merck
	Acetonitrile	Merck
	Ethyl Acetate	Merck
